# Effects of Plasma-Activated Water on Leaf and Fruit Biochemical Composition and Scion Growth in Apple

**DOI:** 10.3390/plants12020385

**Published:** 2023-01-13

**Authors:** Andrei Kuzin, Alexei Solovchenko, Dmitry Khort, Rostislav Filippov, Vladimir Lukanin, Natalya Lukina, Maxim Astashev, Evgeny Konchekov

**Affiliations:** 1 Michurin Federal Scientific Center, 393766 Michurinsk, Russia; 2 Fruit and Vegetable Growing Department, Michurinsk State Agrarian University, 393766 Michurinsk, Russia; 3 Faculty of Biology, Lomonosov Moscow State University, 119234 Moscow, Russia; 4 Federal Scientific Agroengineering Center VIM, 109428 Moscow, Russia; 5 Prokhorov General Physics Institute of the Russian Academy of Sciences, 119991 Moscow, Russia

**Keywords:** plasma-activated water, apple tree, fruit set, yield, mineral nutrient content, bioactive compounds, bench grafting, buds, growth, survival rate

## Abstract

The application of plasma-activated water (PAW) in agriculture has gained the attention of researchers and practitioners. In particular, treatment with PAW is a promising method for increasing scion and rootstock survival as well as augmenting the mineral nutrition applicable to tree fruit crops. However, the applications of PAW are hampered by the lack of information about the effects of PAW on apple tree condition and yield. The increase in survival rate by PAW is believed to stem from the general stimulation of physiological processes in the plant tissue. To assess the actual effect of the PAW treatments, one needs to consider an important indicator of young tree quality such as their vegetative growth. We conducted field experiments to study the possibility of use of PAW for increase in primary nutrient contents in fruits and leaves in an orchard, as well as to assess the scion survival rate and vegetative growth of young grafts in a nursery. The application of PAW influenced the fruitset, yield, leaf nitrogen (N) and potassium (K), fruit phosphorus (P), calcium (Ca) ascorbic acid (AA) and titratable acidity (TA). Treatment with PAW did not significantly reduce the negative impact of the rootstock thickness on the survival rate of bench grafts and their subsequent development. At the same time, scion survival tended to increase in the case when the scions and the rootstocks were of compatible thickness. Further studies of the PAW treatment effects are needed to better understand its applicability in diverse fields of horticulture.

## 1. Introduction

In recent years, studies of so-called “cold plasma” and its derivatives and their application in agriculture have increased [1]. One plasma application in agriculture is stimulation of plants using plasma-activated water (PAW). This is created by exposing water or water solutions to plasma initiated by various types of discharge, for example, direct discharge [2] glow discharge [3], and plasma jet [4]. Chemical reactions initiated by plasma occur in the liquid and at the air-liquid interface that lead to a change in the physicochemical characteristics of liquids. As a result, long-lived reactive oxygen and nitrogen species are formed in liquids, such as hydrogen peroxide, ozone, and ions of nitrogen oxides (NOx^−^). Treatment of biological materials with PAW has similar results to the direct action of CAP [5] but does not have restrictions on the shape of the biological material being processed and has a lower risk to damage to plants. Some interesting results have been obtained in seed treatments with PAW [6]. Foliar treatments with PAW enhanced the content of photosynthetic pigments (chlorophylls) in wheat [7] as well as nitrogen and phosphorus uptake in *Faloppia sachalinensis* [8].

Literature on the application of PAW for foliar treatments, especially in apple, is scarce, although there are ample reports on applications of cold plasma in medicine e.g., for blood coagulation, and disinfection of medical equipment. [9]. In the context of agriculture, the application of PAW directly to fields and orchards is of interest. In the generated PAW, short-living active oxygen and nitrogen species such as hydroxyl- (·OH), nitric oxide- (NO^_^), superoxide (·O_2_^−^) radicals, and ozone (O_3_) are formed, and further react yielding nitric oxide (NO), nitrate (NO_3_^−^) and nitrite (NO_2_^−^), peroxynitrite (ONOO^−^), and hydrogen peroxide (H_2_O_2_) [10,11]. These reports confirmed that PAW can stimulate plant growth [12] and control diseases [13]. However, weekly foliar PAW treatments did not have a significant effect on the accumulation of corn biomass, or on foliar nutrient content [14]. 

Ascorbic acid content is an important feature of fruit quality [15]. PAW treatments of fresh-cut pear fruits had no significant impact on AA content after 8 days of storage [16]. However, PAW treatment of Fuji apples brought about a significant decrease in fruit AA content [17]. Unfortunately, information on the effect of multiple PAW sprayings on growing apple trees on the fruit AA content is still insufficient. There is also a lack of information on PAW impact on apple fruit titratable acidity. To date, there have been mostly discussions of the PAW effect on processed apple products (e.g., juice) in terms of food safety and shelf life. For instance, Tarabová et al. [18] reported that cold plasma treatment insignificantly decreased malic acid content of apple juice and strongly reduced AA concentration.

The above-mentioned investigations were done on annual herbaceous plants, whereas woody plants have been little studies [19,20,21]. Information about the influence of PAW on apple trees is particularly scarce. There are reports about eliminating human pathogens from the apple fruit surface using PAW [22]. To bridge, at least in part, the gap in our knowledge of the effects of PAW on apple trees, two goals were set for this study.

The first goal was to gain additional insights into the using of PAW to augment the effect of traditional mineral fertilizers on apples [23] to reduce their application rates and lower pressure on the environment. The use of foliar fertilizing is currently an important strategy in plant nutrition [24]. Since water solutions of fertilizers are normally used for spraying, it was interesting to test PAW application separately and together with fertilizers. Particular attention was paid to the mineral nutrient content of leaves and fruits of fruit-bearing apple trees, as well as to their yield. 

The second goal was to test the effect of PAW on the outcome of winter grafting. This goal is in line with the growing demand for planting material (young apple trees) for high-density industrial orchards. The deficiency of planting material is a setback for the development of modern apple growing. The quality of apple planting material coming from nurseries determines the potential onset and duration of fruiting in commercial orchards. Bench grafting is frequently used in growing companies during the apple growing season and to provide additional employment for their staff in winter. For winter grafting, the method called bench grafting or improved copulation is normally used [25]. Such a method of grafting is good when the thickness of rootstock and scion is about the same. A sloping cut (the ratio of diameter to length below 1:1.5) is made, followed by a second downward cut on 1/3 of the first cut to prepare the tongue-latch the scion on the rootstock. The two pieces are then fitted together with the latch-tongue interlocking [26].

The scion cutting diameter usually ranges from 6 mm to 9 mm whereas the rootstock diameter sometimes exceeds 14 mm. The producers of plant material buy large batches of rootstocks containing a sizeable number of such overly thick plants. In most cases, they have to discard such plants, increasing their net monetary loss. However, it is possible to use the same technique to graft on overly thick rootstocks provided that the cambial layers are aligned at least on one side of the scion-rootstock coupling [27,28]. We tested the effect of PAW on these partly aligned grafts.

Grafting triggers changes in a plethora of physiological and biochemical processes including biosynthesis of growth regulators and other responses [29]. These processes strongly influence development of the resulting complete fruit trees by modulating scion architecture, and also determine crop productivity and resilience of the tree to stresses such as frost, heat, bacterial and fungal deceases, and pests [30]. The mechanisms causing these effects [31,32,33,34] are expected to overlap, at least partially, with responses to the PAW treatments.

In view of what the above, we tested (i) the influence of PAW on apple tree growth, fruit yield, and primary mineral nutrient contents, and (ii) the effect of PAW on the outcome of winter grafting.

## 2. Results

### 2.1. Experiment #1

#### 2.1.1. Fruitset from Free Pollination and Yield

**Figure 1:**
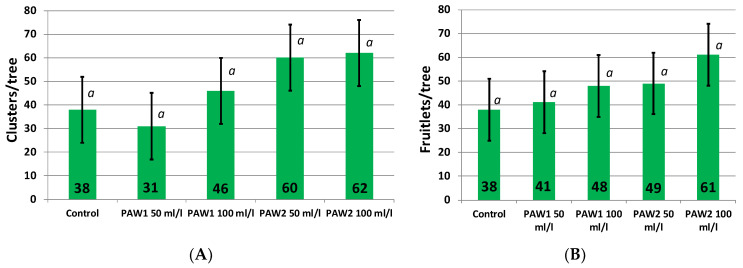
Bloom intensity (clusters/tree) before the PAW treatment (**A**) and abundance of fruitlets on the PAW-treated tress (after June fruitlet drop, (**B**)). Average values are presented. Significantly differing values are marked with different letters. The values significantly differing, according to the Fisher’s LSD criterion (see Section 3), are marked with different letters.

We began the PAW treatments in June, so they could not influence the bloom in the same year and the differentiation and development of flower buds from last year and winter. Nevertheless, a record of inflorescence number was kept for normalization and objective assessment of the effect of PAW on fruiting (Figure 1A). We did not find significant differences between the experimental plots in the number of inflorescences. The largest number of inflorescences was recorded in the plots later treated with PAW2, and the lowest number was in the plot later treated with “PAW1 50 mL/L” plot. 

After flower fertilization, the apple tree forms a large number of small fruits. After several weeks (usually 2–3), the weakest small fruits drop. Three of the PAW sprayings were done before the counting of small fruits. We did not find significant differences between the treatments in the number of small fruits at the end of June after small fruit drop (Figure 1B). The largest number of fruitlets was in the treatment “PAW2 100 mL/L”. The quantity of fruitlets was equal to the number of inflorescences (counted earlier in May) in this treatment. The same result was found in control 1. The number of fruitlets in PAW2 50 mL/L was less than the number of inflorescences, and in the “PAW1 50 mL/L” treatment the number of fruitlets increased. So, we cannot conclude that three PAW sprayings made a significant impact on fruitlet development and drop in June. 

**Figure 2:**
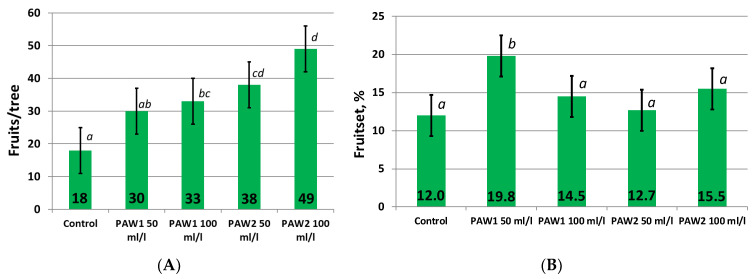
Fruit crop load before harvest (**A**) and fruitset from free pollination (**B**). Average values are presented. The values significantly differing, according to the Fisher’s LSD criterion (see Section 3), are marked with different letters.

Significant differences were recorded in the number of fruits before harvest between some of the treatments (Figure 2A). The largest quantity of fruits was in the “PAW2 100 mL/L” treatment. The number of fruits in “PAW2 50 mL/L” was lower compared to “PAW2 100 mL/L, but it was significantly higher than that in the control. All the other treatments were not significantly different. So, we can see that the fruit drop in July in August in these treatments was not as large as in the others, despite higher crop load. 

The fruit set from free pollination is the ratio of fruits on trees before harvesting to the initial number of flowers, expressed as a percentage. The number of flowers had high variability, and the higher this value in May, the higher the final number of fruits. To make an objective assessment of studied preparation impact on fruit development, we calculated this parameter. The maximum level of fruitset was in the “PAW1 50 mL/L” treatment, and it was significantly higher than that in control 1 (Figure 2B). We did not see significant differences between the other treatments and control 1. 

The highest value of the fruit average mass was recorded in control 1. This value decreased in the PAW treatments (Table 1). Nevertheless, this decrease was significant only in the PAW1 100 mL/L” treatment. The application of the PAW1 did not make a significant impact on the yield compared control 1. The PAW treatments affected significantly the yield compared both to control 1 and the “PAW1” treatment. Interestingly, the yield in PAW2 treatments significantly increased with the increase of PAW working solution concentration. 

#### 2.1.2. The Contents of Primary Nutrients in Apple Leaves

The PAW treatments did not have a clear effect on leaf N content, which indirectly confirmed the high LSD value (Table 2). Perhaps, its high variability was enhanced by heavy rainfall since September 5 till the end of the month (10 days before sampling with total precipitation 39 mm). PAW significantly affected the leaf N status when applied in concentration of 50 mL/L. Using both PAW types in a 50 mL/l concentration did not affect the leaf N content significantly (compared with the control 1).

Foliar P content was relatively low in all treatments. The leaf P status value was also highly variable (see LSD value). The largest foliar P content was in the “PAW2 100 mL/L” treatment, and was significantly higher than that in all other treatments. The remaining experimental PAW treatments were not significantly different from control 1. 

The leaf K content was relatively low. The PAW1 treatments did not influence leaf K. The PAW2 application stimulated an increase in foliar K. The nutrient contents significantly increased with an increase of PAW working solution concentration. 

The highest leaf Ca content was in the leaves of treated with PAW2 (100 mL/L). It was the only treatment where the Ca leaf status was higher than in control 1. We did not see the differences between the other treatments and the control.

#### 2.1.3. Contents of Primary Nutrients in Apple Fruits

Fruit N content had no significant differences in all treatments, including control 1 (Table 3). The lowest fruit N status was in the PAW1 50 mL/L, but it was not significant. The fruit P content significantly increased in PAW1 100 mL/L treatment and was increased in PAW2 treatments. 

Significant increases in fruit K content were noted only in one treatment, PAW1 100 mL/L. PAW treatments had a very positive effect on fruit Ca status. We observed a significant increase in fruit Ca in two treatments: PAW1 100 mL/L and PAW2 50 mL/L, but the fruit Ca status tended to also increase in other experimental treatments.

#### 2.1.4. Biochemical Composition of Fruits

The general trend was represented by an increase of ascorbic acid in fruits under the influence of PAW treatments (Table 4), but only the PAW1 treatments stimulated the increase in AA. The largest amount of AA was found in the PAW1 100 mL/L treatment; it was significantly higher than in other treatments, including the “PAW2 50 mL/L”.

The application of PAW tended to increase monosaccharide contents in harvested mature fruits. This increase was not significant compared to control 1. The highest disaccharide content was in PAW1 100 mL/L. When the working solutions of PAW decreased, the amount of disaccharides also decreased. This disaccharide reduction was significant only in the PAW1 application. 

The PAW treatments stimulated a significant increase in TA except for the PAW2 100 mL/L treatment. We noted that in both PAW types, the increase in working solution concentration led to a decrease in TA.

The fruit dry mass increased after PAW applications in all treatments. The working solution concentrations did not have a significant effect on the dry mass value. RAW1 had some tendency to increase fruit dry mass compared to PAW2 applications.

### 2.2. Experiment #2

#### 2.2.1. Graft Survival Rate

The application of PAW did not influence significantly the bud survival rate (Table 5). The largest number of dead scions was in the treatment with grafting on the “thick” rootstock. It was significantly higher than that in the control and in the treatment with grafting on the “normal” rootstock. PAW did not significantly influence the survival rate difference between bench graft on the “normal” rootstock and the control. The maximum number of completed trees (grafts) with a weak scion development was among the grafts made on the “thick” rootstock. We also did not see a difference between the tree development in the Control and NR+PAW, but in TH+PAW such trees were significantly lower on average. The largest number of well-developed trees was in the Control. In the TR+PAW (the “thick” rootstock), the number of well-developed trees was significantly lower than that grafted on the “normal” rootstock. Overall, the negative effect of the rootstock thickness on the scion survival rate was retained regardless of the PAW treatment. 

**Figure 3:**
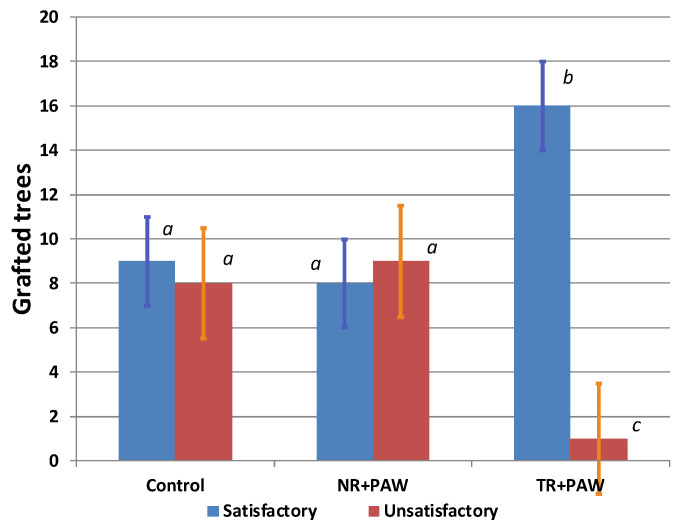
Effect of PAW and rootstock thickness on completed tree development and quality categories. The “satisfactory” category included the trees with “good” and “average” development; the “unsatisfactory” category included “weakly” developed trees and the dead scions (see Table 6). The values significantly differing, according to the Fisher’s LSD criterion (see Section 3), are marked with different letters.

Good maintenance in the nursery makes it possible to get good quality young apple trees even when the completed tree shows average development after grafting. Therefore, we aggregated categories 1 and 2, as well 3 and 4, and considered a further two categories: “satisfactory development” and “unsatisfactory development” (Figure 3).

#### 2.2.2. Growth of the Grafted Trees in Nursery

Young tree growth activity in the nursery is a very important property characterizing their quality. The largest length of the average annual shoot growth was in NR+PAW treatment (Table 6), but the difference from the Control was not considerable. The least annual shoot elongation was in the treatment TR+PAW, and was significantly smaller than in the Control and in NR+PAW. 

## 3. Materials and Methods

### 3.1. PAW Preparation

PAW1 was generated using an electrochemical unit electrolyte vessel with active (Pt) and neutral electrodes. The neutral electrode was made of pyrolityc graphite. The electrodes were connected to a high-frequency (HF) generator (440 kHz). The TRMS current was 0.8 A and the peak current could reach 5 A at the moment of plasma ignition. The operating voltage on the electrodes was 300 V. The short-term power from the generator could reach 1500 W. This was enough to form a vapor-gas bubble on the active electrode and ignite a glow discharge in the vapor phase. A 1% KCl solution was used as the electrolyte. The volume of the experimental reactor was 6 L. Plasma electrolysis of the solution proceeded without the use of a diaphragm. The solution was intensively mixed during electrolysis with a magnetic stirrer. In the reactor operating mode, the correct temperature was no more than 70 °C and was achieved at an average power of 0.8 A × 300 V = 240 W. The duration of PAW synthesis was 6 h. Stable chemical compounds were developed in the solution during this time. The active electrode was slowly destroyed forming carbon and Pt nanoparticles from the manufacturing material (10–20 nm size; 10^12^ pcs/mL concentration). Graphite nanoparticles were not formed, and only Pt nanoparticles were formed. The passive graphite electrode was slowly destroyed during operation and precipitated as a graphite sediment. Nanoparticles were detected and measured using a Zetasizer ULTRA Red Label (USA). The synthesis of nanoparticles was made by electrical erosion of a platinum electrode in a pulsed gas discharge (condensation of the vapor phase of the active electrode material). Therefore, the solution gradually yellowed, and clarified within the next 3–4 weeks with nanoparticles conglomeration and sedimentation. This solution was applied earlier, freshly prepared as a disinfectant and, after a period of time, used for plant irrigation diluted in distilled water (1:200) [35,36]. Some physical and chemical properties of the PAW1 are shown in the Table 7. 

The PAW2 (Table 2) was prepared with a non-contact approach using an argon plasma jet in a nitrogen atmosphere [37]. The PAW2 was also used in the Experiment #2. The apparatus was made based on a technological microwave plasma torch for open-air operation. The microwave source was a commercial magnetron of 1.2 kW power operating at a frequency of 2.45 GHz in continuous generation mode [38,39]. A brightly glowing plasma jet carried ions and argon atoms falling to the surface of the water at high speed. The gas dynamic pressure of the argon plasma created a dip on the distilled water surface ≈1.5 cm deep with a diameter of ≤1.5 cm. The factor acting on the water was jet ultraviolet radiation, which created a halo of photoionization and excitation of the surrounding nitrogen and water vapor.

Redox potential, pH, and electrical conductivity were measured on an S470 SevenExcellence high-precision measuring station (Mettler Toledo, Columbus, OH, USA). Sensor electrodes InLab Expert Pro-ISM and InLab731-ISM (Mettler Toledo) were used. During measurements, aqueous solutions were mixed in laminar mode using a magnetic stirrer (rotation frequency 3 Hz). All measurements were carried out at a solution temperature of 20 ± 1 °C. The number of repetitions was five.

The content of nitrite and nitrate anions in the samples was determined using Griss reagent using a Multiscan FC plate reader (TermoScintific, Vaanta, Finland), and the optical density of the medium was measured at a wavelength of 546 nm. Sodium nitrite and sodium nitrate solutions of known concentration were used for calibration. The number of repetitions was five. 

Concentrations of hydrogen peroxide in aqueous solutions were determined using enhanced chemiluminescence in the luminol-p-iodophenol-horseradish peroxidase system. The luminescence intensity was determined using a Biotox-7A chemiluminometer (ANO ICE, Moscow, Russia). The initial concentration of hydrogen peroxide used for calibration was determined spectrophotometrically at a wavelength of 240 nm with a molar absorption coefficient of 43.6 (M−1 × cm^−1^). The working solution contained 1 mL Tris-HCl buffer pH 8.5, 50 µM p-iodophenol, 50 µM luminol, and 10 nM horseradish peroxidase. Number of repetitions was five.

KCl solution was used to prepare PAW1, and acted as an electrolyte for ignition and maintenance of plasma combustion. Distilled water was used to prepare PAW2. The Pt nanoparticles formed in the first variant were by-products of the synthesis. The effect of Checl nanoparticles on the growth and development of plants in combination with activated water is currently the subject of research by our scientific group.

### 3.2. Location and Conditions of Experiments 

#### 3.2.1. Experiment #1

Experiment #1 was carried out in the experimental orchard of I.V. Michurin Federal Scientific Centre in Michurinsk (52.883805, 40.465342). The objects of the study were apple trees of cv. Ligol grafted on B396 rootstock. The orchard was planted in Autumn 2018, with a planting pattern of 4.5 × 1.2 m. Each treatment included 30 trees in three blocks (10 trees per block). PAW sprayings occurred 10 times during the growing season at an equal time interval from the beginning of June till the middle of September. The fertigation rate on the experiment plots was N_10_P_7_K_12_ in all treatments, including Control1. We reduced the usual fertigation rate to highlight the effect of PAW.

According to the agrochemical survey of spring 2022, the soil of the experimental plot was leached chernozem with low humus content (1.9–2.3%), heavy loamy on sand with pseudo fibers. The depth of the humus horizon was 40–50 cm. The base saturation was 75–86%. The amount of absorbed bases was 24.3–31.2 mg-eq. 100 g^−1^. The upper layers of the soil had a pH_KCl_ of 5.2–5.4. The soil structure was dusty-granular and lumpy-prismatic. The presence of pores in the upper horizons reached 65–70%. Field moisture capacity of the topsoil was 27.3–28.5%. The content of hydrolysable nitrogen in the soil layer at 0–40 cm was 125–135 mg·kg^−1^, and mobile forms of phosphorus 115–123 mg·kg^−1^ and exchangeable potassium were 140–156 mg·kg^−1^ of soil.

Climatic parameters were recorded by an automatic weather station KaipoRain (Russia). Average monthly air temperature in some months was 0.9–4.3 °C, and the season average higher by 0.7 °C than the multiyear average air temperature (Table 8). Exceptions were May and September.

The most spectacular deviations from multiyear averages were in precipitation levels. The amount of rainfall was lower in May and June, very little in August, but in July and especially in September precipitation was higher than usual for these months. The average rainfall was at the same level as the multiyear average. 

#### 3.2.2. Experiment #2

Experiment #2 to study the influence of PAW on bench grafting with different stock diameters was carried out in the Novopavlovskiy district of Stavropol region in Competence Research Center for Fruit Growing and Nursery (44.145225, 42.858708). The study objects were grafts after bench grafting (the scion was from the cultivar Modi^®^, and the rootstock was from M9). We used the cuttings with two buds as the scions. The planting pattern in the nursery was 90 × 20 cm.

### 3.3. Experimental Design and Analysis Methods

Experiment #1 was designed as follows: (1) Control 1 (without PAW treatments); (2) PAW1 50 mL/L; (3) PAW1 100 mL/L; (4) PAW2 50 mL/L, and (5) PAW2 100 mL/L. The amount of working solution sprayed once was 333 mL/tree or 617 L/ha. We made records of blossom cluster number, small fruits at the end of June after fruitlet drop, the number of fruits in one week before harvest, fruit average mass, and yield. We calculated the rate of fruit set from free pollination as the ratio of the number of fruits before harvest to the number of flowers in full bloom (we counted flower in 100 inflorescences and the average value (five) was used as the number of flowers per cluster). We counted flowers in 100 clusters on different trees and average number of flowers was five in the inflorescence. We determined leaf and fruit nitrogen (Kjeldahl method, AKV-20, Russia), phosphorus (molybdenum blue method, Hitachi U-2000, Japan), potassium (flame photometer, FPA-2.01, Russia), and calcium (complexometric method with trilon B) [40]. We also determined the fruit ascorbic acid content (by titration using 2,6-dichlorophenolindophenol solution), fruit titrable acidity (by titrating with 0.1N NaOH solution to pH 8.2, expressed as a percentage of malic acid equivalent), and mono and disaccharides (by the Bertrand method). Fruit dry mass (DM) was assessed gravimetrically.

Experiment#2 design was as follows: (1) Control 2 (grafting on the “normal” rootstock, without PAW treatment); (2) NR (“normal” rootstock—compatible thickness with scion) + PAW—grafting on the “normal” rootstock + PAW treatment, and (3) TR (“thick” rootstock, i.e., too thick compared to scion) +PAW; grafting on the “thick” rootstock with PAW treatment. Each treatment included 51 plants comprising three repetitions (17 plants per replica) in the trial. The bench grafting with dipping of the cuts in PAW was carried out on 21–22 February 2022 following the standard routine of “improved copulation” [25]. After fitting the pieces, the union point was tightly wrapped with grafting tape. Roots were pruned to the length of 18–19 cm, and the upper cut of the scion was dipped for 1 s in grafting wax heated to 66 °C. The grafted trees were padded with wet sawdust and stored in boxes at 1–2 °C in a dark room till planting (approx. 50 days). 

The grafted plants were planted on 16 April 2022, and the survival rate was assessed on 16 May 2022. Depending on scion bud development and new shoot growth, the plants were divided into four categories: with dead scion, with weak shoot development (0.5–4 cm), with an average shoot development (4–6 cm), or with good shoot development (˃6 cm). We also measured new shoot growth in each treatment on 24 plants (8 for each replicate) excluding the dead plants.

### 3.4. Statistical Analysis

Statistical analysis was done using Fisher’s Least Significant Difference (LSD) method. We calculated the smallest significant difference (LSD) at *p* ˂ 0.05. We considered any difference between the average values larger than the calculated LSD_05_ as a significant result.

## 4. Discussion

In our experiments, we tested PAW applications on the efficiency of fruit crop setting. The fruitset from free pollination depends on many factors, among them are pollinating insect efficiency, weather (during fruit bud formation and bloom), and winter hardiness [41]. Therefore, it was necessary to take into account high precipitation levels during bloom that accelerated the end of bloom and decreased the pollinator insect efficiency. These might be the reasons for absence of sizeable effect of triple application of the PAW in June on the number of fruitlets left on trees after the fruitlet drop. The showers in July could also have strongly influenced the fruitlet drop and modulate the effects of the PAW treatment. Since a sharp decrease in the number of fruits in the control was noted after June, one can state that the PAW treatments positively affected the yield due to reducing fruitlet drop. The fruit drop in the “PAW2 100 mL/L” was reduced in June, and in the “PAW1 50 mL/L” in the rest of the preharvest period. Fruit average mass strongly depends on crop load [42]. Therefore, the lowest fruit load was in the control 1 was likely related to relatively high fruit mass in this variant. 

The literature mainly discusses the influence of PAW on seed germination, plant growth, pest and disease control [43]. The complicated process of apple yield formation, which takes two growing seasons and the winter between them, has not been considered in the context of PAW effects. Therefore, there was no reason to expect a significant increase in apple fruit yield as a result of the PAW treatments. The observed positive effect on yield in the PAW2 treatments was quite surprising. We believe that it stemmed mainly from the decrease in fruitlet drop earlier in the growing season.

In principle, PAW can be an alternative source of nitrogen for plants, as indicated by the increase of both foliar nitrate and nitrite [12] due to chemical fixation of atmospheric nitrogen into bioavailable forms by the cold plasma [23]. Still, the significance the nitrogen uptake from PAW by leaves was not confirmed in the experiments [14]. In our study, the leaf N content increased in the PAW1 and PAW2 treatments when the concentration of working solutions was the lowest (50 mL/L). Perhaps, such an effect was due to excessive concentration of different ions in the 100 mL/L PAW working solutions that were inhibitory for the nitrogen metabolism in leaves. 

The low foliar K content could be due to cessation of fertigation three weeks before sampling, and heavy September precipitation, which began 10 days before sampling and could have led to potassium leaching from the leaves [44]. The foliar PAW treatments of maize increased its nitrogen and phosphorus content, but the PAW effect on the other primary nutrient content was not documented [14]. In our study, only the application of PAW2 significantly affected leaf N, K, and Ca contents. The working solution concentration increase from 50 mL/L to 100 mL/L, stimulating significant increases of leaf P and K. The PAW1 application did not cause similar effects on the leaf primary nutrient contents. Likely, the PAW2-specific effects may have stemmed from its stimulatory capacity for the apple tree mineral metabolism. 

People eat fresh and processed fruits all year round [45,46], so the quality and storability of apples are important. The PAW treatments in our study did not significantly affect fruit N contents. Since the overall rate of metabolism in fruits is not as high as in the leaves, the PAW treatments influenced fruits less than leaves. Notably, an increase of P and Ca in the treated fruit was measured. Too high (˃0.09% d.m) as well as too low (˂0.06% d.m.) fruit phosphorus content significantly increases the risk of low temperature breakdown, especially in susceptible cultivars [47]. The fruit P contents in the PAW1 100 mL/l and both PAW2 treatments significantly exceeded this limit. This may have negatively affected fruit storability. The fruit Ca content significantly increased in two treatments (PAW1 100 mL/L and PAW2 50 mL/L) which, in contrast, can be beneficial for fruit storability [48,49,50]. 

Experiment#2 also showed that the application of PAWs did not have a pronounced stimulatory effect on the survival rate of grafts or the growth of the grafts in the nursery. At the same time, published reports state that the influence of PAW is more pronounced on annual herbaceous plants, while we studied the effect PAW on apple, a perennial woody organism. Annual herbaceous plants can have more flexible responses to such treatments. Genetic background, working solution concentrations, handling, and other factors can also influence the responses of plants. For instance, in our previous research we observed an increase of survival rate of cherry and pear graftings with PAW up to 11% compared to non-treated PAW grafts [51].

In the experiments with Arabidopsis, it was found that the use of PAW activated cytosolic calcium, which is an intracellular second messenger, and led to an increase in the activity of various physiological processes [38]. PAW application stimulated seed germination of different crops [52], enhanced the vegetative growth of grape cutting leaves during bud break in laboratory conditions, and increased catalase activity [53]. 

Considering possible negative effect of PAW on biological samples is primarily associated with oxidative stress caused by ROS contained in PAW. We have previously studied the cytotoxic effect of PAW on plant tissues [54]. However, in this study we did encounter a possible cytotoxic effect, although there was no significantly positive effect of PAW on the grafting outcome. Delayed effects of PAW are also possible, and we shall continue monitoring of the experimental plants to pinpoint them should they arise. It should be noted the negative result in the experimental group “TR+PA” (the “thick” rootstock) is likely associated with misalignment of the scion and the rootstock and has little to do with the PAW treatment per se.

Perhaps, the use of PAW will increase the effect of foliar fertilizing when used together in tank mixtures. It has been noted that the foliar spraying PAW increased the electrical conductivity of the roots and the activity of photosynthesis [14]. Therefore, nutrient uptake could be increased because of enhanced physiological activity. We will investigate this in the near future on mature apple trees in orchard. 

## 5. Conclusions

Generally, we observed a slight, but positive effect of the field treatments with PAW 2 on the apple fruit yield, mostly by reducing fruitlet loss. This effect was found in the “PAW2 100 mL/L” treatment (in June) and in the “PAW1 50 mL/L” (throughout the rest of the pre-harvest period).

The foliar N content increased in the treatments with PAW1 and PAW2 at the lowest concentration (50 mL/L), whereas the large concentration of PAW (100 mL/L) could inhibit nitrogen metabolism in leaves. 

The most significant effect on the content of N, K, and Ca in the leaves was seen after the treatment with PAW2. The content of Ca in the fruits increased significantly after two treatments (PAW1 100 mL/L and PAW2 50 mL/L), which may enhance fruit storability, although additional tests are needed to prove this.

We did not observe significant effects of PAW treatment on the outcome of winter grafting, although the yield of the high-quality and good-quality completed trees was higher in the “NR+PAW” than in the control. The PAW treatment did not increase significantly annual shoot elongation. Overall, rootstock thickness and misalignment exerted a more profound effect on the grafting outcome than the PAW treatment. Nevertheless, the PAW-treated plants tended to show higher growth activity under our experimental conditions.

Finally, one cannot rule out delayed PAW effects on tree growth and other parameters studied, so multi-year research on PAW effects is important.

## Figures and Tables

**Table 1:** Impact of PAW on fruit average mass and yield.

Treatments	Fruit Average Mass, g	Yield, t/ha
Control 1	163.5 ^a,^*	7.2 ^a^
PAW1 50 mL/L	142.1 ^a^	7.6 ^a^
PAW1 100 mL/L	130.6 ^b^	8.1 ^a^
PAW2 50 mL/L	150.1 ^a^	10.9 ^b^
PAW2 100 mL/L	146.8 ^a^	13.9 ^b^
LSD_05_ **	25.3	2.7

* Average values are presented. The values significantly differing from the Control 1 are marked with different letters of Latin alphabet. ** Fisher’s Least Significant Difference values (see Section 3). The values significantly differing, according to the Fisher’s LSD criterion (see Section 3), are marked with different letters.

**Table 2:** The effect of PAW on the primary nutrient contents in apple leaves, % d.m.

Treatments	N	P	K	Ca
Control 1	1.49 ^a,^*	0.27 ^a^	0.89 ^a^	1.15 ^a^
PAW1 50 mL/L	2.13 ^b^	0.26 ^a^	0.83 ^a^	1.22 ^a^
PAW1 100 mL/L	1.27 ^a^	0.23 ^a^	0.87 ^a^	1.20 ^a^
PAW2 50 mL/L	2.12 ^b^	0.29 ^a^	1.05 ^b^	1.88 ^b^
PAW2 100 mL/L	1.61 ^a^	0.39 ^b^	1.24 ^b^	1.28 ^a^
LSD_05_ **	0.44	0.09	0.13	0.35

* Average values are presented. The values significantly differing from the Control 1 are marked with different letters. ** Fisher’s Least Significant Difference values (see Section 3). The values significantly differing, according to the Fisher’s LSD criterion (see Section 3), are marked with different letters.

**Table 3:** Effect of PAW on the primary nutrient contents of apple fruits.

Treatments	N	P	K	Ca
Control 1	0,39 ^a,^*	0.082 ^a^	0.66 ^a^	0.0267
PAW1 50 mL/L	0.26 ^a^	0.08 ^a^	0.62 ^a^	0.0333
PAW1 100 mL/L	0.39 ^a^	0.176 ^b^	0.91 ^b^	0.0467 *
PAW2 50 mL/L	0.39 ^a^	0.373 ^c^	0.70 ^a^	0.0667 *
PAW2 100 mL/L	0.37 ^a^	0.229 ^d^	0.63 ^a^	0.0300
LSD_05_ **	0.11	0.040	0.16	0.0096

* Average values are presented. The values significantly differing from the Control 1 are marked with different letters. ** Fisher’s Least Significant Difference values (see Section 3). The values significantly differing, according to the Fisher’s LSD criterion (see Section 3), are marked with different letters.

**Table 4:** Contents of some bioactive compounds in apple fruits.

Treatments	Ascorbic Acid, mg 100 g^−1^	Monosaccharides, % d.m.	Disaccharides, % d.m.	Titratable Acidity, %	Dry Mass (d.m.), %
Control 1	6.24 ^a,^*	6.93 ^a^	2.40 ^a^	1.01 ^a^	14.3 ^a^
PAW1 50 mL/L	7.38 ^b^	7.81 ^a^	1.52 ^b^	1.39 ^c^	16.2 ^b^
PAW1 100 mL/L	8.83 ^c^	7.44 ^a^	1.97 ^b^	1.16 ^b^	16.1 ^b^
PAW2 50 mL/L	6.89 ^a^	7.24 ^a^	1.48 ^b^	1.39 ^c^	15.6 ^b^
PAW2 100 mL/L	6.68 ^a^	7.24 ^a^	1.42 ^b^	0.94 ^a^	15.5 ^b^
LSD_05_ **	1.09	0.97	0.41	0.08	0.9

* Average values are presented. The values significantly differing from the Control 1 are marked with different letters. ** Fisher’s Least Significant Difference values (see Section 3). The values significantly differing, according to the Fisher’s LSD criterion (see Section 3), are marked with different letters.

**Table 5:** Effect of PAW and rootstock thickness on survival rate and development (number of plants).

Treatments	Died Scions	Grafted Tree Development		
		Weak	Average	Good
Control	3 ^a,^*	6 ^a^	3 ^a^	5 ^b^
NR+PAW	2 ^a^	6 ^a^	6 ^b^	3 ^a^
TR+PAW	5 ^b^	11 ^b^	1 ^a^	0 ^a^
LSD_05_ **	2	3	2	1

* Average values are presented. The significantly differing values are marked with different letters. ** Fisher’s Least Significant Difference values (see Section 3). The values significantly differing, according to the Fisher’s LSD criterion (see Section 3), are marked with different letters.

**Table 6:** Effect of PAW and rootstock thickness on completed tree’s annual shoot growth, cm.

Treatments	Average Annual Shoot Elongation	Cumulative Annual Shoot Elongation
Control 2	85.6 ^a,^*	862.7 ^a^
NR+PAW	86.7 ^a^	880.7 ^a^
TR+PAW	80.5 ^b^	722.3 ^c^
LSD_05_ **	5.5	76.7

* Average values are presented. The significantly differing values are marked with different letters. ** Fisher’s Least Significant Difference values (see Section 3). The values significantly differing, according to the Fisher’s LSD criterion (see Section 3), are marked with different letters.

**Table 7:** Physicochemical properties of the PAW.

PAW Type	Exposure Time, min	Electrical Conductivity, mS/cm	pH	Redox, μV	NO_3_^−^, mM	H_2_O_2_, mM
PAW1	360	24.9 ± 1.2	8.3 ± 0.2	598 ± 26	22.05 ± 0.98	7.12 ± 0.68
PAW2	240	14.0 ± 1.0	4.5 ± 0.2	560 ± 18	87.00 ± 5.00	0.11 ± 0.01

**Table 8:** Average monthly air temperatures (°C) and total monthly precipitation (mm) during the growing season.

Month	Temperature, °C	Precipitation, mm
April	9.5	52.4
May	11.9	44.8
June	20.6	47.2
July	21.8	79.4
August	23.7	23.0
September	11.7	121.0
October	7.9	43.0
Mean IV–X	15.3	58.7

## Data Availability

The data are available from the corresponding author on reasonable request.
